# MYC-regulated lncRNA NEAT1 promotes B cell proliferation and lymphomagenesis via the miR-34b-5p-GLI1 pathway in diffuse large B-cell lymphoma

**DOI:** 10.1186/s12935-020-1158-6

**Published:** 2020-03-19

**Authors:** Chong-Sheng Qian, Ling-Jie Li, Hai-Wen Huang, Hai-Fei Yang, De-Pei Wu

**Affiliations:** 1 Department of Hematology, The First Affiliated Hospital of Soochow University, No. 188, Shizi Street, Suzhou, 215006 People’s Republic of China; 2 Jiangsu Institute of Hematology, The First Affiliated Hospital of Soochow University, Suzhou, 215006 People’s Republic of China; 3 Institute of Blood and Marrow Transplantation, Suzhou, 215006 People’s Republic of China; 4 Department of HLA Laboratory, Jiangsu Institute of Hematology, The First Affiliated Hospital of Soochow University, Suzhou, 215006 People’s Republic of China

**Keywords:** Cell proliferation, Diffuse large B-cell lymphoma, GLI1, MYC, LncRNA NEAT1

## Abstract

**Background:**

LncRNA NEAT1 has been identified as a tumour driver in many human cancers. However, the underlying mechanism of lncRNA NEAT1 in diffuse large B-cell lymphoma (DLBCL) progression is unclear.

**Methods:**

The expression levels of NEAT1, GLI1 and miR-34b-5p were detected by RT-qPCR and Western blotting in DLBCL tissues and cell lines. MTT and colony formation assays were performed to examine cell proliferation, while annexin-V staining and TUNEL assays were performed to measure cell apoptosis. The effect of NEAT1, GLI1 and miR-34b-5p on cell cycle-associated proteins was evaluated by Western blotting. Dual-luciferase reporter and RNA immunoprecipitation (RIP) assays were employed to investigate the interaction between NEAT1 and miR-34b-5p or GLI1 and miR-34b-5p. Moreover, chromatin immunoprecipitation (ChIP) was performed to demonstrate the interaction between MYC and NEAT1.

**Results:**

NEAT1 and GLI1 were upregulated while miR-34b-5p was downregulated in DLBCL tissues and cell lines compared to normal controls. Knockdown of NEAT1 or overexpression of miR-34b-5p inhibited cell proliferation but promoted cell apoptosis. Overexpression of NEAT1 reversed GLI1-knockdown induced attenuation of cell proliferation. In other words, NEAT1 acted as a competing endogenous RNA (ceRNA), regulating the miR-34b-5p-GLI1 axis, further affecting the proliferation of DLBCL. Moreover, MYC modulated NEAT1 transcription by directly binding to the NEAT1 promoter.

**Conclusion:**

We revealed that MYC-regulated NEAT1 promoted DLBCL proliferation via the miR-34b-5p-GLI1 pathway, which could provide a novel therapeutic target for DLBCL.

## Background

Lymphoma is a type of malignant cancer that occurs worldwide, contributing 4% of the total number of new cancer cases diagnosed in 2018. Non-Hodgkin lymphoma (NHL) is the most common subtype of lymphoma and mainly includes diffuse large B-cell lymphoma (DLBCL). DLBCL is aggressive and heterogeneous, and approximately 75% of DLBCL patients are defined as Ann Arbor stage III or IV [1, 2]. Emerging evidence indicates large roles for lncRNAs in malignant B cells; in these cells, lncRNAs can influence oncogenic signaling as well as the response to clinical treatments [3–5]. For example, aberrant expression of lncRNA NEAT1 is found in DLBCL tissues and is often associated with disease progression and poor prognosis [6].

The GLI1 oncogene has been implicated in the pathobiology of DLBCL [7–9]. Agarwal et al. identified GLI1 as providing insights into the contribution of Hedgehog signaling in the pathobiology of malignant tumours [7]. GLI1 contributes to the cell survival of DLBCL through the expression of AKT in DLBCL and likely in other malignant tumours. Active IKKβ promotes GLI1 expression, leading to the increased cell viability of DLBCL in vivo and in vitro [8]. Sun et al. found that GLI1 inhibition repressed cell growth and cell cycle progression and promoted apoptosis as well as autophagy depending on ERK1/2 activity in human chondrosarcoma cells [9].

MicroRNAs (miRNAs) are endogenous ∼ 22 nt RNAs that can play important regulatory roles in animals and plants by targeting mRNAs for translational repression [10]. The targeting of miRNAs could be a novel therapeutic approach, as evidenced by tumour regression in mouse models and initial promising data from clinical trials [11–14]. One recent study showed that miR-101, upregulated in DLBCL, suppressed DLBCL cell proliferation and facilitated apoptosis by inhibiting the expression of MEK1 [15], while miR-155, which is downregulated in DLBCL, suppressed DLBCL cell proliferation and facilitated apoptosis by upregulating SOCS3 expression to suppress the JAK-STAT3 signaling pathway [16]. Thus, miRNAs may play different roles through various signaling pathways. In our study, we observed that miR-34b-5p was downregulated in DLBCL and that a targeting relationship existed between miR-34b-5p and GLI1 according to TargetScan analysis. Moreover, the interaction between NEAT1 and miR-34b-5p was predicted by StarBase, indicating that the NEAT1-miR-34b-5p-GLI1 axis might function in DLBCL progression.

With the development of microarray technology and immunohistochemistry, DLBCL has been classified into germinal centre B cell-like (GCB) DLBCL and activated B cell-like (ABC) DLBCL based on gene expression profiling studies. The GCB DLBCL samples expressed genes that are characteristic of normal germinal centre B cells, but ABC DLBCL samples had genes characteristic of plasma cells [17]. In addition to GCB DLBCL and ABC DLBCL subtypes, double-hit lymphomas that had concurrent chromosomal rearrangements of MYC plus BCL2 or BCL6 were considered aggressive DLBCL. MYC, BCL2 and BCL6 are the most common oncogenes in DLBCL. A study showed that MYC rearrangements were found in 12.2% of DLBCL, with 17.7% in GCB DLBCL and 6.5% in ABC DLBCL, and these rearrangements indicated a poor prognosis after standard combination chemotherapy [18]. MYC rearrangements plus BCL2 rearrangements (4.8%) were observed in GCB DLBCL, and MYC rearrangements with BCL6 rearrangements (1.2%) were also detected. Although many studies have mainly focused on the effect of MYC and BCL2 rearrangements, it is also recognized that MYC and BCL2 can be induced in other ways. High expression of MYC and BCL2 or BCL6 was significantly associated with poor prognosis and survival [19, 20].

MYC is a master transcriptional regulator that controls almost all cellular processes [21–23]. To be exact, it can promote cell activation, growth and proliferation while concomitantly sensitizing cells to apoptosis [24]. MYC-related microRNAs can regulate DLBCL progression via core cellular pathways [25]. Recently, it was shown that the Smurf2-YY1 axis regulates MYC expression to reduce B cell proliferation [26]. In addition, MYC can bind to NEAT1 and inhibit its expression to regulate cell apoptosis in chronic myeloid leukaemia (CML) [27]. Thus, we predicted that MYC could bind to the promoter of NEAT1 by JASPAR analysis and modulate the expression of NEAT1. Taken together, we hypothesized that MYC may participate in the regulation of the NEAT1-miR-34b-5p-GLI1 axis, further investigating DLBCL pathogenesis.

In this study, we mainly focused on clarifying the mechanism by which MYC regulates the cell proliferation of DLBCL via the NEAT1-miR-34b-5p-GLI1 signaling axis, which might provide novel targets for DLBCL therapies.

## Methods

### Cell lines and cell transfection

The DLBCL cell lines OCI-Ly1, OCI-Ly8, OCI-Ly10 and SUDHL-4 were obtained from the Cell Bank of Type Culture Collection, Chinese Academy of Science (Shanghai, China). OCI-Ly1 was established from the bone marrow of a 44-year-old male with stage 4B B-cell non-Hodgkin lymphoma (B-NHL; diffuse large cell) at relapse in 1983. OCI-Ly8 was established from a 58-year-old male with diffuse large B-cell lymphoma. OCI-Ly10 was established from a 66-year-old female with DLBCL. SUDHL-4 was established from a 38-year-old male with DLBCL.

OCI-Ly1, OCI-Ly8, and OCI-Ly10 cells were grown in 90% Iscove’s medium with 10% foetal bovine serum (FBS) and supplemented with penicillin G and streptomycin, while SUDHL-4 cells were grown in 90% RPMI-1640 medium with 10% FBS and supplemented with penicillin G and streptomycin, l-glutamine, and HEPES. We performed Mycoplasma sp. and other contaminant tests every 3 months.

Cells were transfected with shRNAs targeting NEAT1 or GLI1, expression vectors containing full-length NEAT1 or MYC, or miR-34b-5p mimic, which were purchased from GenePharma Co., Ltd. (Shanghai, China) using Lipofectamine 2000 (Invitrogen, Carlsbad, USA) according to the manufacturer’s instructions. Cells were harvested 48 h after transfection.

### Human samples

Sixty samples (healthy control, n = 30; DLBCL, n = 30) were provided by the Department of Hematology, The First Affiliated Hospital of Soochow University. Patient organization and case access were in line with the “Guidelines for the Diagnosis and Treatment of Diffuse Large B-Cell Lymphoma in China” (2013 Edition). All DLBCL patients were newly diagnosed, patients with other tumours were excluded, and patients had no serious impairment of heart, lung, brain, liver or kidney function. There were 12 males and 18 females with an average age of 54.26 ± 6.24 years. In terms of Ann Arbor staging, 17 cases were stage I–II (early stage) and 13 cases were stage III–IV (progressive stage). All samples were approved by the Ethics Committee of the First Affiliated Hospital of Soochow University. Written informed consent was signed by all enrolled patients.

### 3-(4,5-Dimethyl-2-thiazolyl)-2,5-diphenyl-2-H-tetrazolium bromide (MTT) assay

Cells were seeded in 96-well plates at 5 × 10^3^ cells/well. After the indicated number of days (0–3 days), cell viability was determined by MTT (Sigma, USA). The cell viability in each well was measured in terms of optical density (OD) at wavelength 490 nm by the use of a microplate reader (Bio-Rad Laboratories, Hercules, CA, USA). Every sample was measured in triplicate.

### Flow cytometry

Apoptotic cells were analyzed by an annexin V staining kit (Thermo Fisher Scientific). Briefly, the cells were harvested and washed twice with cold PBS. The cells were resuspended in 1× binding buffer and stained with annexin V-FITC reagent for 15 min at room temperature. Then, the cells were washed with 1× binding buffer and incubated with propidium iodide (PI) for 5 min on ice. The stained cells were analysed by flow cytometry with a BD FACSCalibur cytometer (BD Biosciences, San Diego, CA, USA).

### Colony formation assay

Cells were seeded at a low density (0.4 × 10^3^ cells/well) in 6-well plates and cultured for 6 days. After 6 days of culture, colonies were washed with PBS, fixed with 10% formaldehyde for 5 min and stained with 1% crystal violet for 30 s. The images of each well were captured, and the number of colonies containing at least 50 cells in each well was counted.

### Terminal deoxynucleotidyl transferase dUTP nick end labelling (TUNEL) staining assay

Apoptotic cells were evaluated using the In Situ Cell Death Detection Kit (Roche, Basel, Switzerland, Germany) according to the manufacturer’s instructions. Nuclei were stained with DAPI. The images were captured by fluorescence microscopy (Leica Microsystems GmbH, Wetzlar, Germany). The percentage of apoptotic cells in each view was counted.

### Reverse transcription-quantitative real-time polymerase chain reaction (RT-qPCR)

Total RNA was extracted from cells using TRIzol reagent (Invitrogen, Thermo Fisher Scientific) following the manufacturer’s instructions. cDNA synthesis was performed with the Prime-Script RT-PCR master mix (Takara). SYBR Green Premix Ex Taq (Takara) was used for quantitative RT-PCR analysis. The primers for genes of interest are listed: NEAT1 (forward: 5′-GAGTTAAGGCGCCATCCTCA-3′ and reverse: 5′-AGCACTGCCACCTGGAAAAT-3′), GLI1 (forward: 5′-GCCAATC ACAAATCAGTCTCC-3′ and reverse: 5′-TGCTCCTAACCT GCCCAC-3′), GAPDH (forward: 5′-CCAGGTGGTCTCCTCTGA-3′ and reverse: 5′-GCTGTAGCCAAATCGTTGT-3′), miR-34b-5p (forward: 5′-GTCGTATCCAGTGCAGGGTCCGAGGTATTCGCACTGGATACGAC CAATCA-3′ and reverse: 5′-GCCTAGGCAGTGTCATTAGC-3′), U6 (forward: 5′-CTCGCTTCGGCAGCACA-3′ and reverse: 5′-AACGCTTCACGAATTTGCGT-3′), and MYC (forward: 5′-CGACGAGACCTTCATCAAAAAC-3′ and reverse: 5′-CTTCTC TGAGACGAGCTTGG-3′). Each sample was measured in duplicate, and each experiment was repeated three times.

### Western blot analysis

DLBCL cells were collected, washed with cold PBS buffer, and lysed on ice for 30 min using lysis buffer (RIPA buffer, 89900, Thermo Fisher). Proteins were harvested from the lysates, and protein concentrations were quantified. Then, equal amounts of proteins from each sample were loaded into SDS–polyacrylamide gels and transferred to a polyvinylidene difluoride (PVDF) membrane. The membrane was blocked with 5% skim milk for 1 h at room temperature (RT). Next, the membrane was incubated with primary antibodies at 4 °C overnight. Primary antibodies against GLI1 (ab151796), cyclin D1 (ab226977), CDK4 (137675), p27 (ab45872), MYC (ab39688) and β-actin (ab8227) were purchased from Abcam. Antibodies against GAPDH were purchased from ProteinTech (Chicago, USA). The membrane was then incubated with horseradish peroxidase (HRP)-conjugated secondary antibody for 1 h at RT. The bands were detected with ECL reagent purchased from Millipore Corp.

### Dual-luciferase reporter assay

The wild-type or mutant 3′-UTR region of NEAT1 or GLI1 predicted to bind to miR-34b-5p was cloned downstream of a firefly luciferase reporter in the pmirGLO vector. The mixture of reporter vector, control vector and miR-34b-5p was transfected into DLBCL cell lines OCI-Ly1 or SUDHL-4. The luciferase activity was assessed using a Dual-Glo^®^ Luciferase kit according to the manufacturer’s instructions.

### RNA immunoprecipitation (RIP) assay

RIP experiments were performed using the Magna RIP™ RNA-Binding Protein Immunoprecipitation Kit (Millipore, USA) according to the manufacturer’s protocol. Lysates from DLBCL cell lines OCI-Ly1 or SUDHL-4 were incubated with anti-NEAT1 or anti-GLI1 antibody for 4 h at 4 °C. Co-precipitated RNAs were analysed by qRT-PCR.

### Chromatin immunoprecipitation (ChIP) assay

ChIP experiments were performed with the EZ-Magna ChIP™ Chromatin immunoprecipitation kit (Millipore, USA). Briefly, DLBCL cell lines OCI-Ly1 or SUDHL-4 were cross-linked with 1% formaldehyde for 10 min at room temperature. Cells were then lysed and sonicated in lysis buffer to obtain chromatin fragments. Next, the resulting fragments were extracted by incubation with anti-MYC antibody (anti-IgG antibody as a negative control) based on the manufacturer’s protocol. Co-precipitated DNAs were analysed by RT-qPCR.

### Statistical analysis

All data are presented as the mean of at least triplicate samples ± standard deviation. The data were analysed with GraphPad Prism (GraphPad Software, San Diego, CA). Statistical analysis was performed with SPSS 22.0 (SPSS Inc., Chicago, USA). Student’s t-test was used to evaluate differences between two groups. P values smaller than 0.05 (*P < 0.05, **P < 0.01, ***P < 0.001) were considered statistically significant.

## Results

### LncRNAs NEAT1 and GLI1 were upregulated while miR-34b-5p was downregulated in DLBCL tissues and cell lines

**Fig. 1:**
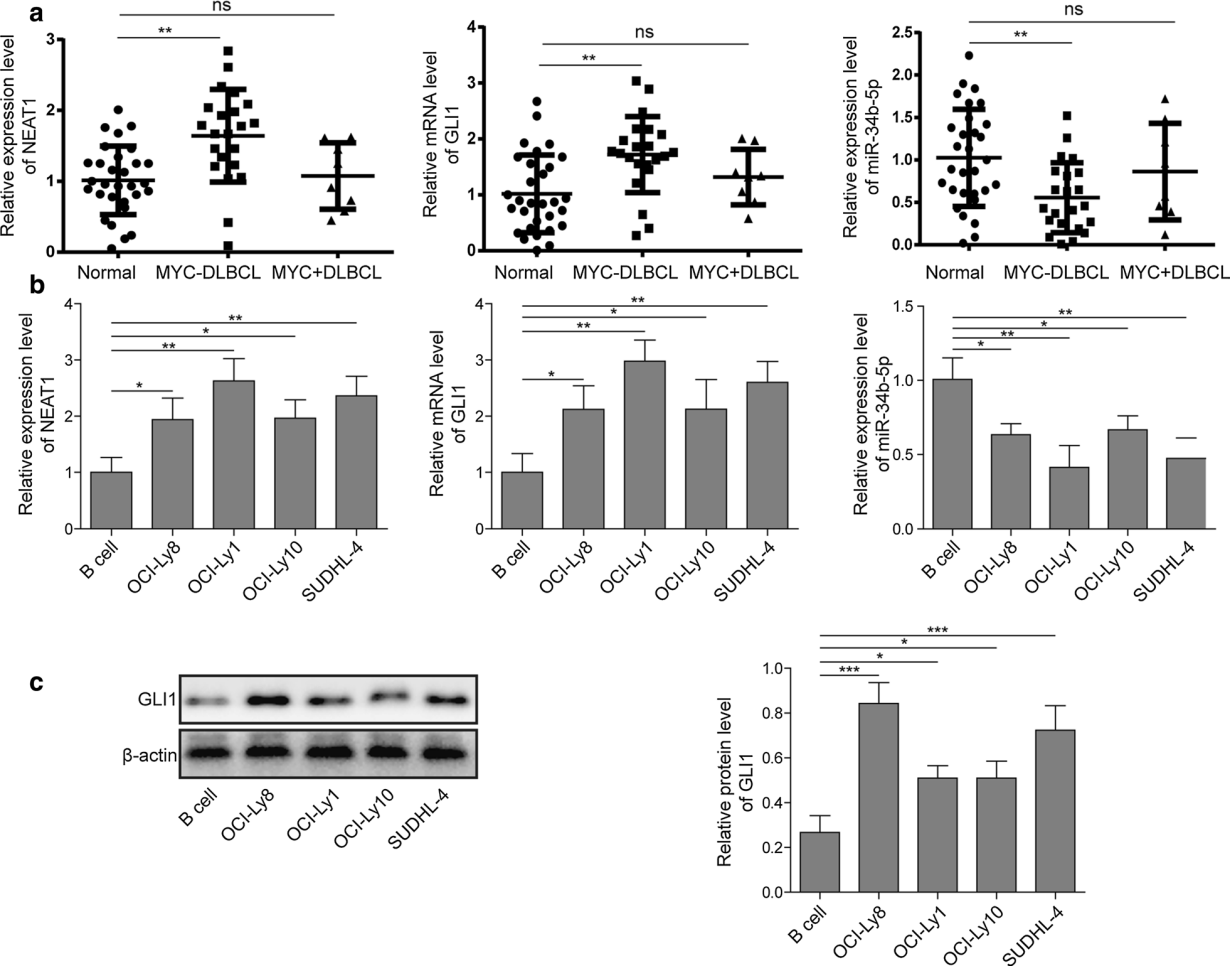
LncRNAs NEAT1 and GLI1 were upregulated while miR-34b-5p was downregulated in DLBCL tissues and cell lines. **a** The levels of NEAT1, GLI1 and miR-34b-5p were detected by RT-qPCR in tissues from DLBCL patients with MYC rearrangement (n = 8), patients without MYC rearrangement (n = 22) or healthy donors (n = 30). **P < 0.01 vs. normal, T-test. **b** The levels of NEAT1, GLI1 and miR-34b-5p were measured by RT-qPCR in the DLBCL cell lines OCI-Ly1, OCI-Ly8, OCI-Ly10, SUDHL-4 and in normal B cells. *P < 0.05 and **P < 0.01 vs. normal B cells, T-test. **c** Immunoblotting and quantification of GLI1 (and β-actin as loading control) in DLBCL cell lines OCI-Ly1, OCI-Ly8, OCI-Ly10, SUDHL-4 and in normal B cells. *P < 0.05 and ***P < 0.001 vs. normal B cells, T-test

To investigate the role of lncRNA NEAT and GLI1 in DLBCL, we first measured their expression in MYC-positive DLBCL patients with or without MYC rearrangement and in normal samples by RT-qPCR. The mRNA levels of NEAT1, GLI1 and miR-34-5p were not significantly different between DLBCL with MYC rearrangement (n = 8) and normal tissues (n = 30); however, there was an obvious increase in NEAT1 and GLI1 mRNA levels and a dramatic decrease in miR-34-5p mRNA levels in DLBCL without MYC rearrangement (n = 22) compared to normal tissues (n = 30) (Fig. 1a). These results indicated that MYC rearrangement may affect the prognosis of DLBCL, but the expression of MYC protein is also related to the development of DLBCL by other mechanisms. MYC rearrangement and tumorigenicity of MYC protein exhibit the same trends, but the expression of MYC protein is not necessarily related to MYC gene rearrangement, so they may participate in two different mechanisms to influence the prognosis of DLBCL.

In other words, the mRNA levels of NEAT1 and GLI1 were also examined in 4 different DLBCL cell lines and normal B cells by RT-qPCR, and they were significantly upregulated in these 4 DLBCL cell lines, especially in OCI-Ly1 and SUDHL-4, when compared with normal B cells (Fig. 1b, c). We also evaluated miR-34b-5p expression, and a reduction in expression was demonstrated in these 4 DLBCL cell lines, with a more dramatic drop in OCI-Ly1 and SUDHL-4 cells. Moreover, consistent with transcriptional expression, the protein level of GLI1 was enhanced in DLBCL cell lines compared to normal B cells, especially in the OCI-Ly8 and SUDHL-4 cell lines (Fig. 1c). Therefore, our findings suggested that aberrant elevation of NEAT1 and GLI1 and decreased expression of miR-34b-5p might play an important role in the regulation of DLBCL.

### Knockdown of lncRNA NEAT1 suppressed cell proliferation and facilitated cell apoptosis in DLBCL

**Fig. 2:**
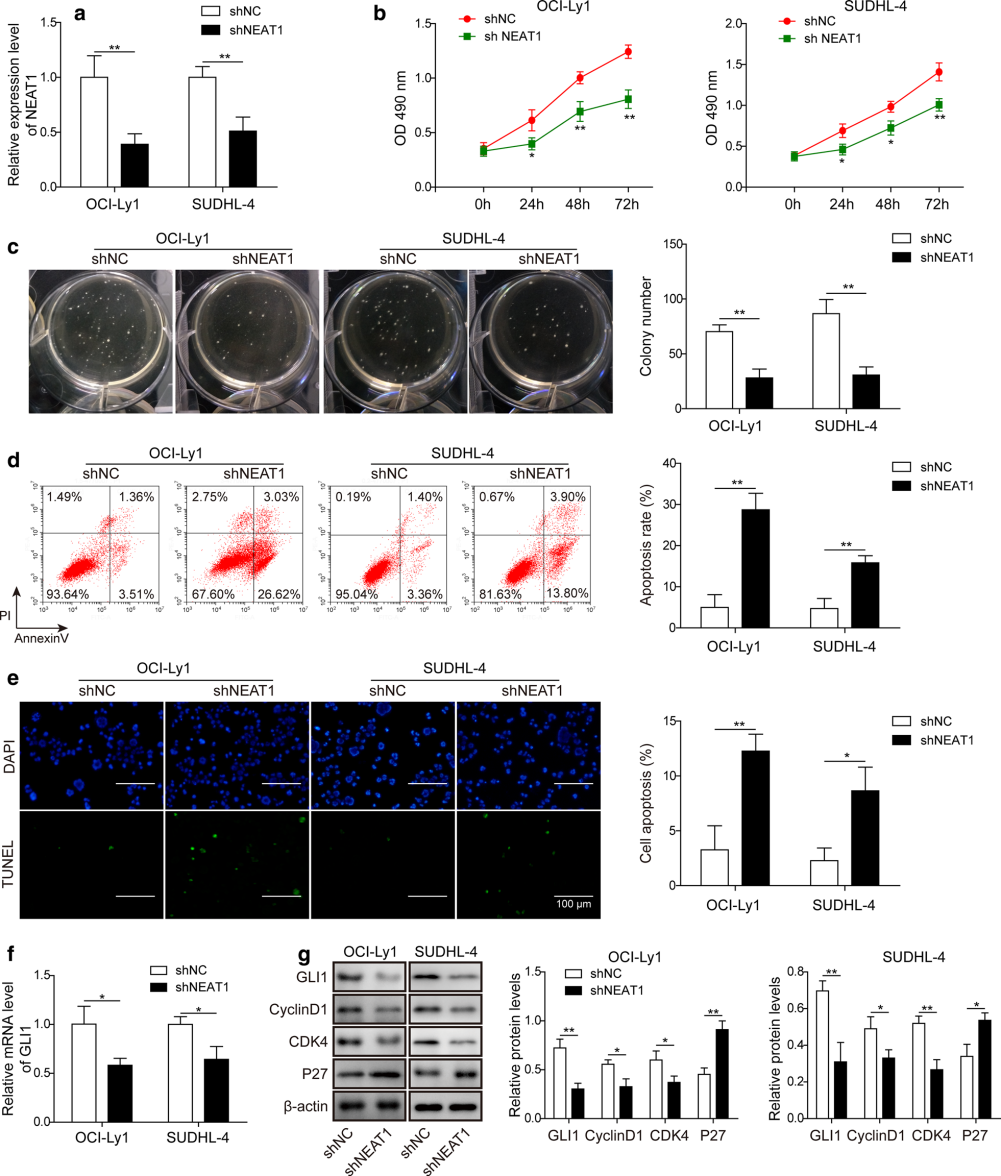
Knockdown of lncRNA NEAT1 suppressed cell proliferation and facilitated cell apoptosis in DLBCL. **a** RT-qPCR for NEAT1 expression in NEAT1 knockdown cells (shNEAT1) or negative control cells (shNC). **P < 0.01, ns = not significant vs. shNC, T-test. **b** Cell viability was measured in NEAT1 knockdown cells (shNEAT1) or negative control cells (shNC) with MTT assay. *P < 0.05, **P < 0.01 vs. shNC, T-test. **c** Colony formation assays were performed in NEAT1 knockdown cells (shNEAT1) or negative control cells (shNC). A representative image of the colony formation assay in OCI-Ly1 and SUDHL-4 cells is shown (left), and the total number of colonies per plate was counted (right). **P < 0.01 vs. shNC, T-test. **d** Flow cytometry analysis of annexin-V/PI staining of apoptotic cells following NEAT1 knockdown in OCI-Ly1 and SUDHL-4 cell lines. A representative image of FACS staining in OCI-Ly1 and SUDHL-4 cells is shown on the left, and the statistical data are shown on the right. **P < 0.01 vs. shNC, T-test. **e** TUNEL staining for OCI-Ly1 and SUDHL-4 shNEAT1 cell lines and their shNC lines. Representative images of TUNEL staining (left) and the statistical data (right) are shown. *P < 0.05, **P < 0.01 vs. shNC, T-test. **f** The mRNA level of GLI1 was assessed in OCI-Ly1 and SUDHL-4 shNEAT1 cell lines and their shNC lines by RT-qPCR. *P < 0.05 vs. shNC, T-test. **g** Immunoblotting and quantification of GLI1, cyclin D1, CDK4 and p27 (and β-actin as loading control) in OCI-Ly1 and SUDHL-4 shNEAT1 cell lines and their shNC lines. *P < 0.05, **P < 0.01 vs. shNC, T-test

To further investigate the function of NEAT1 in DLBCL cells, we constructed NEAT1 knockdown OCI-Ly1 and SUDHL-4 cells using the corresponding shRNAs. The efficiency of NEAT1 knockdown was confirmed by RT-qPCR, and the NEAT1 levels were significantly reduced after transfection with shNEAT1 (Fig. 2a). The MTT assay was employed to measure cell proliferation, and the results showed that compared to shNC control transfection, knockdown of shNEAT1 effectively decreased the growth of OCI-Ly1 and SUDHL-4 cells (Fig. 2b). Moreover, colony formation assays displayed marked suppression of colony formation after knockdown of NEAT1 (Fig. 2c). Then, annexin-V-FITC/PI staining and TUNEL staining were used to measure the apoptosis of OCI-Ly1 and SUDHL-4 cells, which was facilitated by knockdown of NEAT1 (Fig. 2d, e). In addition, cyclin D1, as an important marker of proliferation, was reported to be a target of GLI1 [28]. To investigate possible signalling pathways involved in NEAT1-mediated cell proliferation effects, we used Western blotting to measure the level of GLI1 pathway activation by assessing GLI1, cyclin D1, cyclin D1-dependent kinase 4 (CDK4) and p27 expression. The results showed that knockdown of NEAT1 markedly suppressed the expression of proliferation-related proteins, indicating that NEAT1 knockdown might exert a suppressive effect on the proliferation of DLBCL cells through GLI1 (Fig. 2f, g). This evidence collectively suggests that knockdown of NEAT1 suppresses cell proliferation and promotes cell apoptosis in DLBCL.

### Overexpression of miR-34b-5p attenuated cell proliferation and accelerated cell apoptosis in DLBCL in vitro

**Fig. 3:**
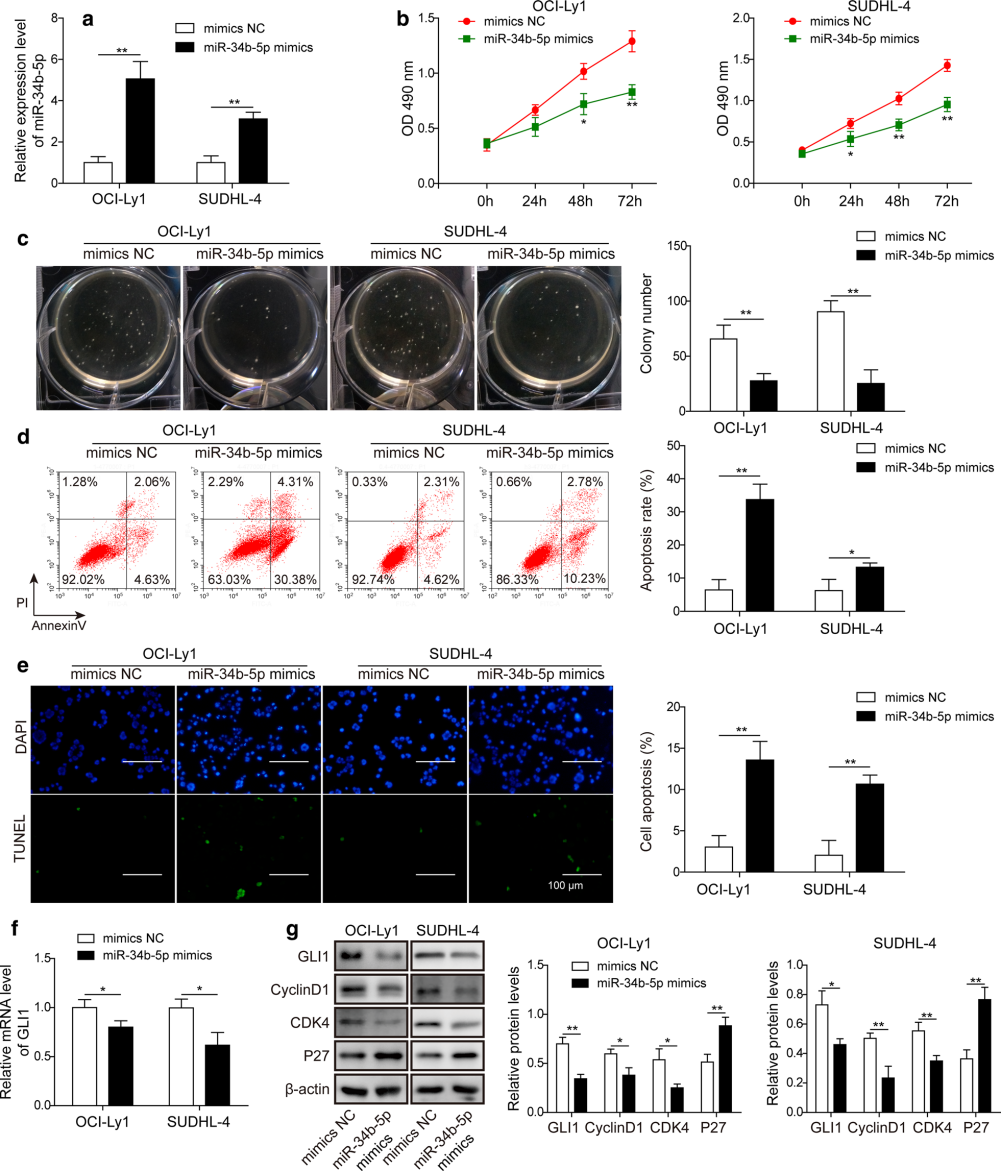
Overexpression of miR-34b-5p attenuated cell proliferation and accelerated cell apoptosis in DLBCL in vitro. **a** The miR-34b-5p levels were measured by RT-qPCR after miR-34b-5p mimic or mimic NC transfection into OCI-Ly1 and SUDHL-4 cell lines. **P < 0.01 vs. mimic NC, T-test. **b** Cell viability was detected by MTT assay in OCI-Ly1 and SUDHL-4 cell lines transfected with miR-34b-5p mimic or mimic NC. *P < 0.05, **P < 0.01 vs. mimic NC, T-test. **c** Colony formation assays were performed in OCI-Ly1 and SUDHL-4 cell lines transfected with miR-34b-5p mimic or mimic NC. A representative image (left) is shown, and the total number of colonies per plate were counted (right). **P < 0.01 vs. mimic NC, T-test. **d** Flow cytometry analysis of apoptotic cells by annexin-V/PI staining following miR-34b-5p mimic or mimic NC in OCI-Ly1 and SUDHL-4 cell lines. A representative image of FACS staining is shown on the left, and the statistical data are shown on the right. *P < 0.05 and **P < 0.01 vs. mimic NC, T-test. **e** TUNEL staining in miR-34b-5p mimic or mimic NC. A representative image of TUNEL staining (left) and the statistical data (right) are shown. **P < 0.01 vs. mimic NC, T-test. **f** The mRNA level of GLI1 was assessed by RT-qPCR in OCI-Ly1 and SUDHL-4 lines transfected with miR-34b-5p mimic or mimic NC. *P < 0.05 vs. mimic NC, T-test. **g** Immunoblotting and quantification of GLI1, Cyclin D1, CDK4, p27 (and β-actin as loading control) in OCI-Ly1 and SUDHL-4 cells transfected with miR-34b-5p mimic or mimic NC. *P < 0.05, **P < 0.01, vs. mimic NC. T-test

To demonstrate the contribution of miR-34b-5p to DLBCL progression, we transfected a negative control mimic (NC mimic) or miR-34b-5p mimic into OCI-Ly1 and SUDHL-4 cells and found that significantly higher levels of miR-34b-5p were observed, as suggested by overexpression of miR-34b-5p (Fig. 3a). Cell proliferation was measured by MTT assay, and the results showed that compared to cells with NC mimic, cells with miR-34b-5p mimic presented significantly slower growth (Fig. 3b). Similar results were obtained when examining the colony formation of OCI-Ly1 and SUDHL-4 cells. The results revealed a significant decrease in colony numbers after overexpression of miR-34b-5p (Fig. 3c). As suggested by the induction of cell apoptosis in NEAT1 knockdown DLBCL cells, we also found that cell apoptosis was markedly enhanced in OCI-Ly1 and SUDHL-4 cells harbouring higher levels of miR-34b-5p by annexin-V-FITC/PI staining and TUNEL assay (Fig. 2d, e). Finally, we measured the effect of NEAT1 on GLI1 and proliferation-related proteins, including cyclin D1, CDK4 and p27, and found that decreased GLI1, cyclin D1 and CDK4 expression accompanied by increased p27 expression was present after miR-34b-5p overexpression (Fig. 2g), indicating that miR-34b-5p might suppress DLBCL cell proliferation.

### Overexpression of lncRNA NEAT1 reversed GLI1 knockdown-mediated suppression of cell proliferation

**Fig. 4:**
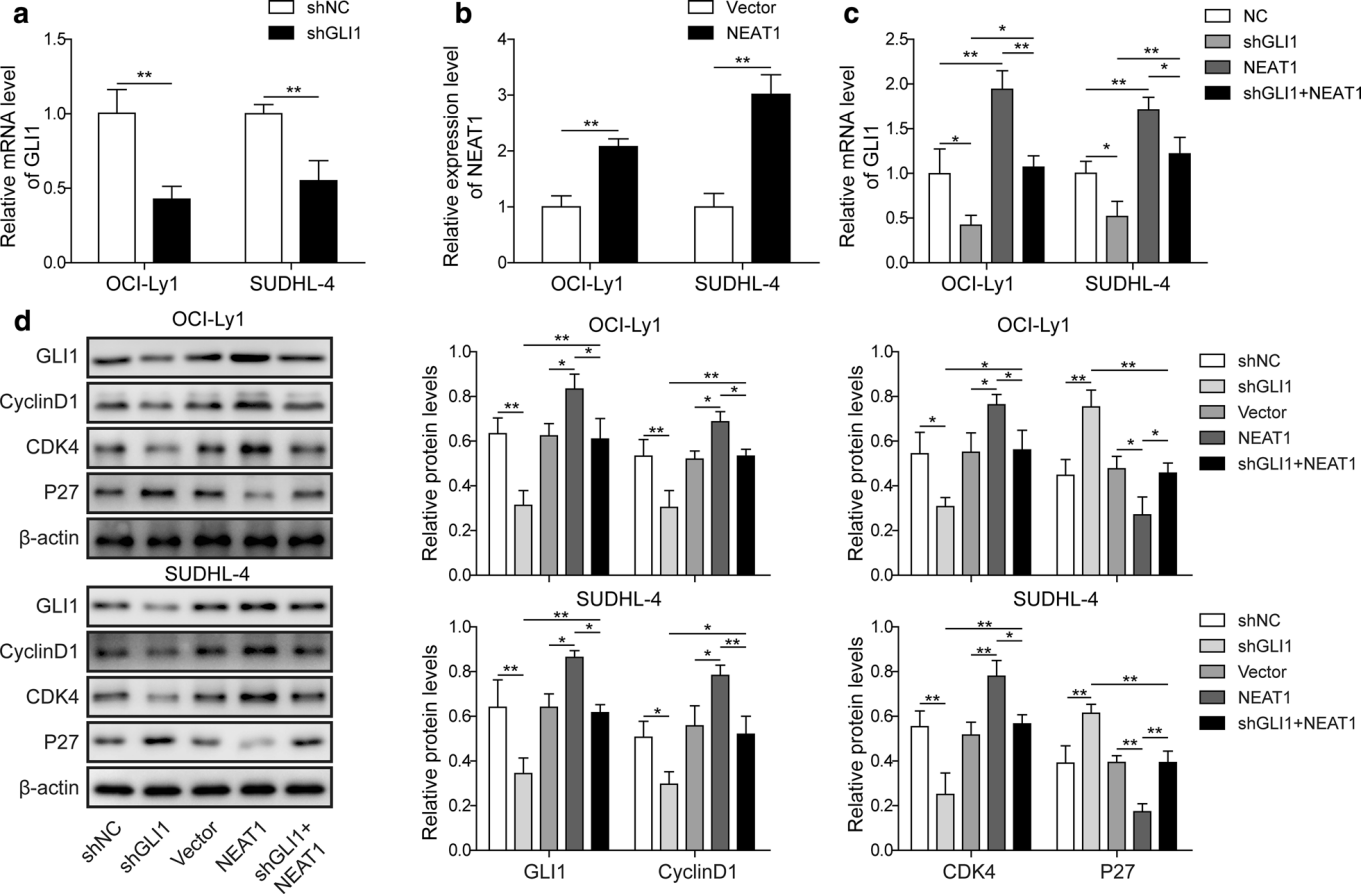
Overexpression of lncRNA NEAT1 reverses the suppression of cell proliferation by knockdown of GLI1. **a** RT-qPCR to determine GLI1 expression in GLI1 knockdown cells (shGLI1) or negative control cells (shNC). **P < 0.01 vs. shNC, T-test. **b** RT-qPCR to determine NEAT1 expression in NEAT1-overexpressing cells (NEAT1) or negative control cells (Vector). **P < 0.01 vs. Vector, T-test. **c** RT-qPCR for GLI1 expression in GLI1 knockdown cells (shGLI1), NEAT1 overexpression cells (NEAT1), GLI1 knockdown plus NEAT1 overexpression cells (shGLI1 + NEAT1) and negative control cells (NC). *P < 0.05, **P < 0.01, T-test. **d** Immunoblotting for GLI1, Cyclin D1, CDK4 and p27 (and β-actin as loading control) in the cell lines described in **c**. The quantification of each protein is shown. *P < 0.05, **P < 0.01, T-test

To investigate whether GLI1 is involved in NEAT1-mediated cell proliferation, we constructed GLI1 knockdown (shNC and shGLI1) and/or NEAT1 overexpression (vector and NEAT1) OCI-Ly1 and SUDHL-4 cells. Compared to shNC cells, the GLI1 level was markedly reduced after shGLI1 treatment, while a significantly higher level was observed after overexpression of NEAT1 (Fig. 4a, b). We further used RT-qPCR to measure the mRNA level of GLI1 and found that the GLI1 level was rescued by NEAT1 overexpression after GLI1 knockdown, as validated by Western blotting (Fig. 4c, d). In addition, we also detected proliferation-related proteins, such as cyclin D1, CDK4 and p27, and the results showed that the decrease in cyclin D1 and CDK4 expression after GLI1 knockdown was reversed by NEAT1 overexpression, while the induction of p27 after GLI1 knockdown was rescued by NEAT1 overexpression. In summary, these results suggested that GLI1 could be the vital downstream regulator of NEAT1 that mediates the promotion of cell proliferation.

### LncRNA NEAT1 acted as ceRNA, sponging miR-34b-5p to regulate GLI1 expression

**Fig. 5:**
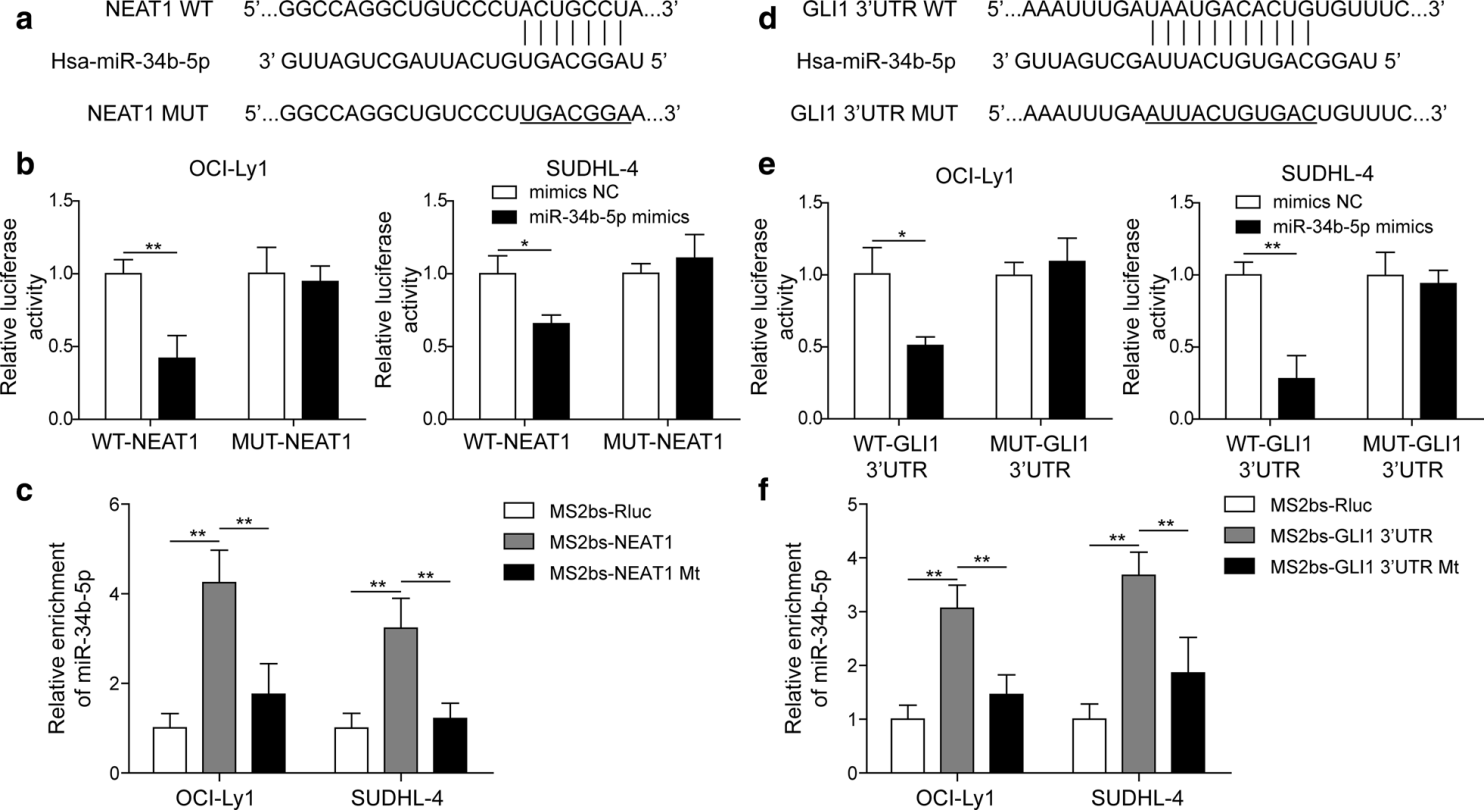
LncRNA NEAT1 acted as ceRNA, sponging miR-34b-5p to regulate GLI1 expression. **a** Diagram showing that StarBase predicts the interaction between lncRNA NEAT1 and miR-34b-5p. **b** The effect of wt-NEAT1 or mut-NEAT1 on miR-34b-5p expression detected by dual-luciferase reporter assay. *P < 0.05, **P < 0.01, vs. Control, T-test. **c** Interaction between wt-NEAT1 or mut-NEAT1 and miR-34b-5p was detected by RIP. **P < 0.01, vs. MS2bs-Rluc or MS2bs-NEAT1 Mt, T-test. **d** TargetScan predicts the binding region between miR-34b-5p and the GLI1 3′-UTR. **e** Dual-luciferase reporter assay was performed to analyse the influence of miR-34b-5p on wt-GLI1 3′-UTR or mut-GLI1 3′-UTR. *P < 0.05, **P < 0.01, vs. Control, T-test. **f** Interaction between miR-34b-5p and GLI1 3′-UTR was detected by RIP. **P < 0.01, vs. MS2bs-Rluc or mut-GLI1 3′-UTR Mt, T-test

Computer-based miR target detection programs to predict the binding sites of NEAT1 and miR-34b-5p (http://starbase.sysu.edu.cn/) and miR-34b-5p and GLI1 (http://www.targetscan.org/) were performed, and the results are shown in Fig. 5a, d. To determine whether NEAT1 could bind to miR-34b-5p, a dual-luciferase reporter assay was performed using vectors constructed with NEAT1 sequences with recognition sites or recognition sites mutated in the presence of miR-34b-5p mimic. The results showed that miR-34b-5p suppressed the luciferase activity of wild-type (wt-) NEAT1 but not mutant (mut-) NEAT1 (Fig. 5b). Moreover, RIP assay revealed that miR-34b-5p was significantly enriched by wt-NEAT1, and the enrichment was markedly reduced on mut-NEAT1 (Fig. 5c). In addition, to evaluate whether GLI1 could bind to miR-34b-5p, a dual-luciferase reporter assay was carried out using vectors constructed with GLI1 3′-UTR sequences with recognition sites or recognition sites mutated in the presence of miR-34b-5p mimic. The results showed that decreases in the signal normalized to the firefly luciferase signal were observed upon miR-34b-5p mimic and pmirGLO-GLI1 3′-UTR-wt transfection (Fig. 5e). In addition, miR-34b-5p was obviously enriched in the wt-GLI1 3′-UTR but not in the mut-GLI1 3′-UTR (Fig. 5f). These results indicated that NEAT1 might act as a ceRNA, modulating GLI1 expression by sponging miR-34b-5p.

### MYC modulated DLBCL proliferation through regulating NEAT1 transcription by binding to the promoter

**Fig. 6:**
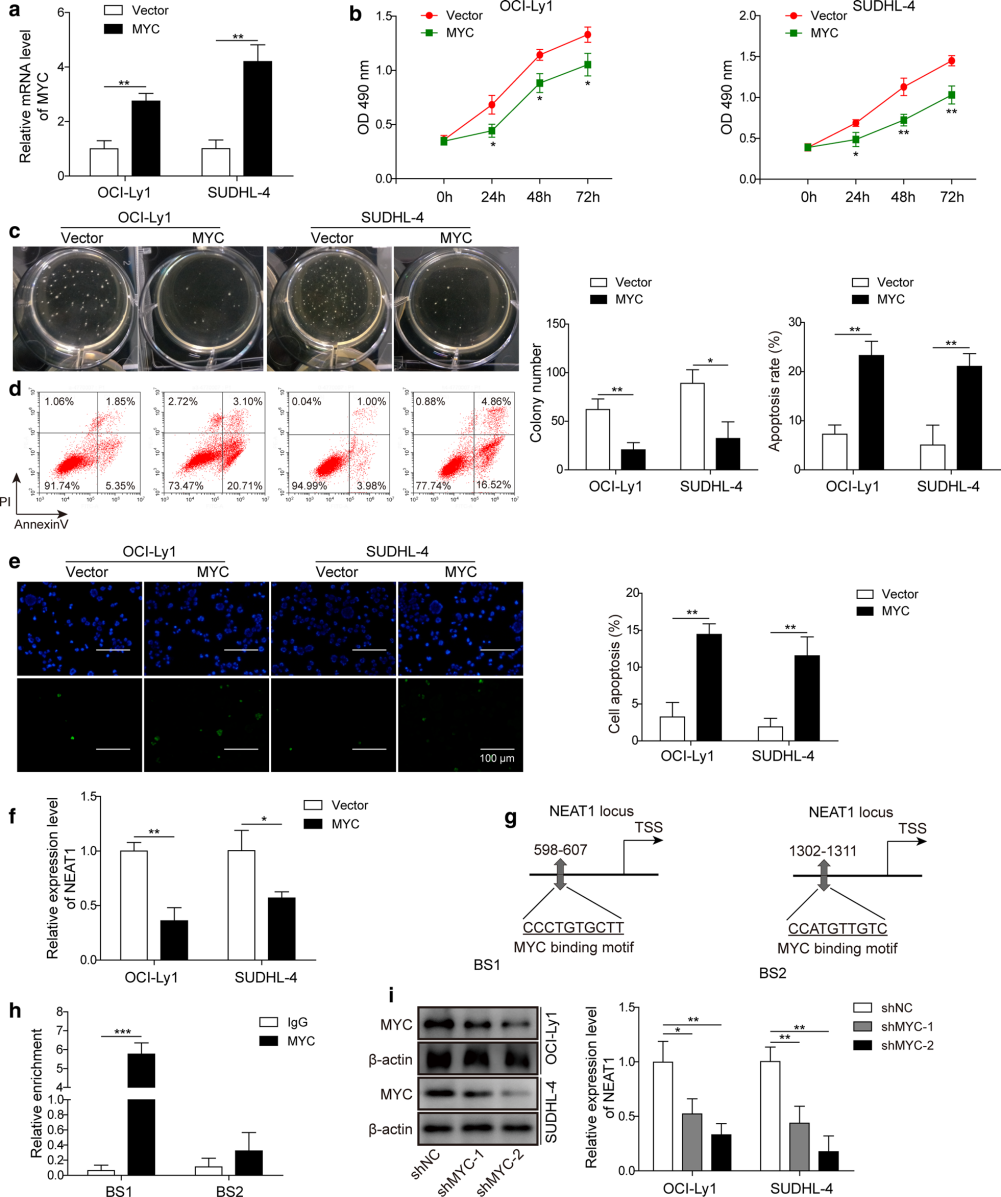
MYC modulated DLBCL proliferation through regulating NEAT1 transcription by binding to the promoter. **a** RT-qPCR to determine MYC expression in MYC overexpression cells (MYC) or negative control cells (Vector). **P < 0.01, vs. Vector, T-test. **b** MTT assay to determine cell proliferation in MYC-overexpressing cells (MYC) or negative control cells (Vector). *P < 0.05, **P < 0.01, vs. Vector, T-test. **c** Colony formation assay was performed in MYC-overexpressing cells (MYC) or negative control cells (Vector). A representative image of the colony formation assay in OCI-Ly1 and SUDHL-4 cells is shown (left), and the total number of colonies per plate was counted (right). *P < 0.05, **P < 0.01, vs. Vector, T-test. **d** Flow cytometry analysis of apoptotic cells by annexin-V/PI staining following MYC overexpression in OCI-Ly1 and SUDHL-4 cell lines. A representative image of FACS staining is shown on the left, and the statistical data are shown on the right. **P < 0.01 vs. Vector, T-test. **e** TUNEL staining in MYC-overexpressing cells. A representative image of TUNEL staining (left) and statistical data (right) are shown. **P < 0.01 vs. Vector, T-test. **f** RT-qPCR to determine NEAT1 expression in MYC-overexpressing cells (MYC) or negative control cells (Vector). *P < 0.05, **P < 0.01, vs. Vector, T-test. **g** Known or potential binding sites of MYC on the promoter of NEAT1 were examined by JASPAR. **h** CHIP assay was performed to detect the interaction of MYC on BS1 or BS2. ***P < 0.001, vs. IgG, T-test. **i** Western blot to determine MYC levels (left panel) and ChIP assay to determine enrichment of MYC on the promoter of NEAT1 (right panel) were performed in MYC knockdown cell lines (shMYC-1, shMYC-2) and negative control cell lines (shNC); *P < 0.05, **P < 0.01, vs. shNC, T-test

A previous study suggested that MYC can bind directly to the lncRNA NEAT1 promoter, reducing its expression to promote cell apoptosis in chronic myeloid leukaemia (CML) [27]. Moreover, the binding sites of MYC in the NEAT1 promoter region were predicted by bioinformatics analysis using JASPAR (http://jaspar.genereg.net/). To measure whether MYC exerted a similar effect in DLBCL, we successfully constructed MYC-overexpressing OCI-Ly1 and SUDHL-4 cell lines, and higher levels of MYC were detected in MYC-overexpressing cells than control cells by RT-qPCR (Fig. 6a). The MTT assay (Fig. 6b) and colony formation assay (Fig. 6c) demonstrated that MYC overexpression attenuated the viability and colony formation ability of OCI-Ly1 and SUDHL-4 cells. In addition, overexpression of MYC promoted the apoptosis of OCI-Ly1 and SUDHL-4 cells, and cells harbouring higher MYC displayed more apoptosis by annexin-V staining (Fig. 6d) and TUNEL staining (Fig. 6e). Next, we detected NEAT1 expression modulated by MYC, and as expected, the mRNA level of NEAT1 was suppressed by MYC overexpression (Fig. 6f). JASPAR analysis predicted two MYC binding sites, BS1 and BS2, on the NEAT1 promoter (Fig. 6g), and ChIP assay results showed that MYC could bind to BS1 but not to BS2 (Fig. 6h). Finally, NEAT1 expression was measured after MYC knockdown, and a marked increase was observed in response to MYC knockdown (Fig. 6i). This evidence collectively suggested that MYC could directly bind to the NEAT1 promoter to regulate its expression, further affecting DLBCL proliferation.

## Discussion

It is now widely understood that mutations within noncoding regions of the genome are major determinants of human diseases such as cancers [29–31]. Long noncoding RNAs (lncRNAs) are functionally defined as transcripts containing > 200 nucleotides in length that have no protein coding potential, and many lncRNAs are uniquely expressed in specific cancer types [32–34]. For example, lncRNA NEAT1 was identified as a potential prognostic predictor in glioma [35]. Aberrant lncRNA NEAT1 expression was also found in DLBCL tissues and was associated with disease progression and poor prognosis [6]. However, the underlying mechanisms remain largely unclear. In this study, we examined the expression of NEAT1, miR-34b-5p and GLI1 in DLBCL cell lines. The results revealed significantly higher levels of NEAT1 and GLI1 and lower levels of miR-34b-5p in DLBCL cell lines than in normal B cells. These data suggested that NEAT1, miR-34b-5p and GLI1 might be jointly involved in DLBCL growth. There are two major subtypes of DLBCL: activated B-cell (ABC) and germinal centre B-cell (GCB) [36]. The ABC subtype is clearly associated with poor survival when treated with standard chemoimmunotherapy. However, the associations between ABC/GCB and NEAT1 or GLI1 need to be investigated further.

Next, we performed MTT and colony formation assays and found that knockdown of NEAT1 or overexpression of miR-34b-5p suppressed cell proliferation. Moreover, an increase in cell apoptosis was observed after knockdown of NEAT1 or overexpression of miR-34b-5p in DLBCL cell lines by annexin-V staining and TUNEL assay. In addition, overexpression of NEAT1 rescued the GLI1 knockdown-induced attenuation of cell proliferation, indicating that NEAT1 functioned as an oncogene via GLI1 in DLBCL. The binding sites of NEAT1 and miR-34b-5p were predicted by StarBase, and those of miR-34b-5p and GLI1 were predicted by TargetScan. The results were also validated by dual-luciferase reporter and RIP assays, indicating that the NEAT1-miR-34b-5p-GLI1 axis exerted a vital effect on DLBCL progression.

MYC has been shown to bind to the promoter of NEAT1, and suppression of NEAT1 expression regulates cell apoptosis in chronic myeloid leukaemia (CML) [27]. GLI1 inhibition has been reported to repress cell growth and cell cycle progression and promote apoptosis in human chondrosarcoma cells [9]. The proteins p27, CDK4, and cyclin D1 are associated with the cell cycle and proliferation [37–39]. Thus, we first determined the correlation between GLI1 and p27/CDK4/cyclin D1. Here, we also found that overexpression of MYC could repress DLBCL cell proliferation and promote cell apoptosis. Moreover, MYC inhibited NEAT1 transcription in DLBCL cell lines via the direct binding of MYC to the NEAT1 promoter. Considering the complexity of MYC, these results seem to be contradictory to the results of some studies, but they are also reasonable because the outcome depends on the level of MYC overexpression. A high level of MYC overexpression could induce apoptosis despite the promotion of cell proliferation, but a low level of MYC induced lymphomagenesis, suggesting that apoptosis antagonizes MYC oncogenic activity. It is not surprising that MYC overexpression negatively regulated NEAT1 expression, while low MYC positively modulated NEAT1, which facilitated the proliferation of DLBCL, which agreed with a previous study by others. However, the dual regulation MYC could occur through many different mechanisms to affect proliferation and apoptosis. MYC deregulation has been linked to the activation of tumour suppressor p53, and p53 mutations are one of most frequently detected mutations in DLBCL. Moreover, the consequence of MYC-induced apoptosis was rescued either by overexpression of anti-apoptotic BCL-2 family proteins or by a lack of pro-apoptotic proteins promoting MYC-induced lymphomagenesis.

## Conclusion

Overall, this work revealed the NEAT1-miR-34b-5p-GLI1 axis as a modulator of DLBCL progression. MYC directly bound to the NEAT1 promoter to regulate the NEAT1 level. Knockdown of NEAT1 restrained cell proliferation and facilitated DLBCL cell apoptosis of DLBCL through the miR-34b-5p-GLI1 pathway. This work established the foundation for further mechanistic studies and provided novel targets for antitumour therapeutics in DLBCL.

## Data Availability

All data generated or analyzed during this study are included in this published article.

## Abbreviations

DLBCL: Diffuse large B-cell lymphoma
LncRNA NEAT1: NEAT1
CHIP: Chromatin immunoprecipitation
RIP: RNA immunoprecipitation
NHL: Non-Hodgkin lymphoma
